# Master S-N curve method to fatigue damage assessment of the high-ply cutter body

**DOI:** 10.1038/s41598-024-82716-w

**Published:** 2024-12-30

**Authors:** Wenfei Liu, Bo Chen, Chao Zhou, Wei Li, Hengji Yu

**Affiliations:** 1 College of information engineering, Zhejiang University of technology, Hangzhou, 310023 China; 2 School of Intelligent Manufacture, Taizhou University, Taizhou, 318000 China; 3 Jack technology Co.,Ltd, Taizhou, 318000 China

**Keywords:** Master S-N curve, Equivalent structural stress, Fatigue damage assessment, Finite element simulation, High-ply cutter, Mechanical engineering, Mathematics and computing

## Abstract

At present, the cutting fabric thickness of the high-ply cutter can reach 100 mm, which requires high vacuum pressure resistance performance, and the fatigue reliability of the cutter body is an important indicator of cutting performance. In this paper, the cutter body was analyzed by finite element simulation, the equivalent structural stress and Von Mises stress at key positions were calculated separately. Then, the master S-N curve method and traditional method were used to evaluate fatigue damages at key locations of the cutter body, which are compared and analyzed with the fatigue damage of measured dynamic stress. The results show that the maximum fatigue damage calculated by the master S-N curve method is 0.223, which is the safest. it verifies the effectiveness and reliability of the master S-N curve method in evaluating the fatigue damage of the cutter body. Meanwhile, the fatigue damage of the three measuring points calculated by the three methods is all below 0.5, and it indicates that the design of the cutter body is reasonable. In addition, the master S-N curve method and equivalent structural stress method were first applied to the fatigue damage assessment of high-ply cutter body, which provides an efficient and high-precision approach for the anti-fatigue design of cutter body.

## Introduction

High-ply and intelligent cutter is a high-tech product that organically integrates mechatronics technology, computer control technology, artificial intelligence control technology, network information technology, etc. It is widely used for cutting leather, specialized fabrics, and special fabrics in aviation, aerospace, military, automotive, clothing, and other industries. The maximum thickness of the fabric cut by the high-ply cutter can reach 100 mm, and the adsorption vacuum pressure is high. Therefore, the cutter body needs to have good pressure bearing function. In addition, the cost of high-ply cutter is high, and their body is a large welded structural component, the fatigue reliability of the body directly affects the overall performance and economic benefits.

The high-ply cutter body belongs to large welded structural components, and the weak positions of large welded structural components often appear in the weld toe area of the welded joint, especially in the weld at the stiffness mutation, which is more prone to fatigue cracks^1–4^. At present, there are many studies on the fatigue strength evaluation methods of welded joints, and the most commonly used method is still the nominal stress method recommended by various national standards, which calculates the fatigue strength of welded components by combining it with S-N curves of different grades of welded joints^5–8^. The BS standard^9^and IIW standard^10^provide rich fatigue resistance design data for steel and aluminum alloy welded joints; The AAR standard^11^provides a large amount of fatigue design data for steel welded joints. Although the relevant parameters and data provided by each standard consider the influence of residual stress, they do not separately provide the gradient effect of stress concentration on the welded joint^12–17^. Moreover, due to the geometric discontinuity of welded joints, it is difficult to obtain the nominal stress and elastic stress concentration coefficient at the weld joint from the finite element model^18–20^. In addition, due to the fact that the evaluation of weld grade is not only related to the geometric shape of the joint, but also to the load mode of the welded part, there is a certain subjectivity in selecting the S-N curve of the welded joint to calculate fatigue damage^21–24^.

In view of the above analysis, hot spot stress method uses linear extrapolation to solve the hot spot stress value at the weld toe of the welded joint^25^, and this method effectively solves the problem of weld toes being difficult to solve due to geometric discontinuities in welded joints^26–28^. However, in finite element analysis, this hot spot stress value is affected by the element type and mesh size^29^. Dong Pingsha^30^proposed a fatigue strength evaluation method based on the combination of equivalent structural stress and principal S-N curve. This new method is given from the perspective of force balance, so stress is not affected by the size and type of elements. This method has been widely used for fatigue life assessment of products such as aviation^31,32^, railway vehicles^33,34^, and ships^35,36^, etc^37,38^. Meanwhile, it has been included in ASME standards^39^. However, this method has not yet been applied to the fatigue strength assessment of high-ply cutter.

In order to provide a highly accurate fatigue damage assessment method for the cutter body and a reference for the anti-fatigue design of the high-ply cutter. In this paper, based on the characteristics of high negative pressure and high requirements for the rigidity and strength of high-ply cutter, the finite element model of the cutter body with the contour of the weld is established and simulated by finite element method. Then, according to the finite element simulation results, the positions of stiffness mutation are determined, and the equivalent structural stress at that positions are calculated. Finally, traditional fatigue damage assessment methods, master S-N curve method, and dynamic stress test method were used to evaluate the fatigue damage of key positions on the cutter body, and the fatigue damage results were compared and analyzed.

## Theoretical background

### Structural stress method

Due to the gap in the weld toe, the stress distribution of the weld along the thickness direction is nonlinear, so it is difficult to solve the stress distribution directly. This nonlinear stress was decomposed into structural stress and notch stress in literature^40^. Although the notch stress contains the nonlinear part, it is independent of the external force and is in self-equilibrium. However, the structural stress is only related to external forces and does not consider the influence of nonlinear stress. Its value is the sum of membrane stress and bending stress, and the mathematical expression is

1 $${\sigma _s}={\sigma _m}+{\sigma _b}$$

Where ${\sigma _s}$ is the structural stress, ${\sigma _m}$ and ${\sigma _b}$ are the membrane stresses and bending stresses along the plate thickness, respectively.

When the plate thickness is given, the uniformly distributed membrane stress and the bending stress in the section are:

2 $$\left\{ \begin{gathered} {\sigma _m}=\frac{{{f_y}}}{t} \hfill \\ {\sigma _{\text{b}}}=\frac{{6{m_x}}}{{{t^2}}} \hfill \\ \end{gathered} \right.$$

Where *t* is the plate thickness, ${f_y}$ is the y-direction line force of the unit welding wire, ${m_x}$ is the line moment around the x-axis.

Bring formula (2) into formula (1), the equation can be obtained

3 $${\sigma _s}=\frac{{{f_y}}}{t}+\frac{{6{m_x}}}{{{t^2}}}$$

**Fig. 1:**
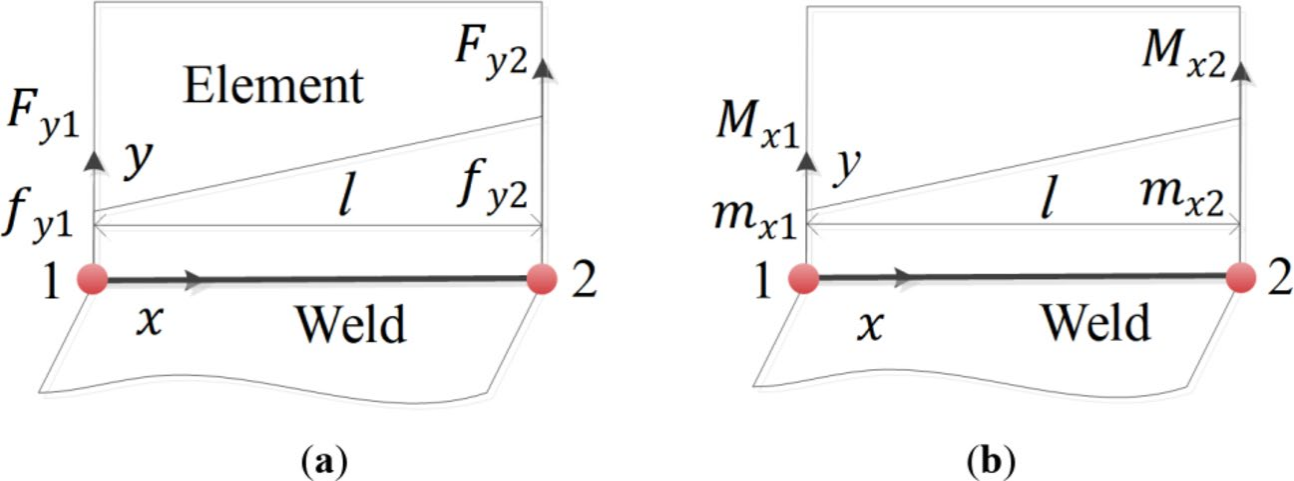
Schematic representation of the line forces and line moments for individual element (**a**) line force and (**b**) line moment.

In the finite element simulation analysis, the distributed load on the element edge is converted to the node, and the structural stress method needs to transform the nodal force and moment obtained by the finite element into line force and line moment. When the equivalent structural stress method is applied to the plate-shell finite element model, the stress state of individual element is shown in Fig. 1.

Where *l* is the length of individual element, ${F_{y1}}$ and ${F_{y2}}$ are the nodal forces of nodes 1 and 2 in the y-direction, ${f_{y1}}$ and ${f_{y2}}$ are the line forces of nodes 1 and 2 in the y-direction, ${M_{x1}}$ and ${M_{x2}}$ are the line moments of nodes 1 and 2 around the x-axis, ${M_{x1}}$ and ${M_{x2}}$ are the line moments of nodes 1 and 2 around the *x*-axis.

According to Fig. 1, the force balance equation and moment balance equation are established from the force state of the element:

4 $$\left\{ \begin{gathered} {F_{y1}} \hfill \\ {F_{y2}} \hfill \\ \end{gathered} \right\}=\left[ \begin{gathered} \frac{l}{3}\;\;\;\frac{l}{6} \hfill \\ \frac{l}{6}\;\;\;\frac{l}{3} \hfill \\ \end{gathered} \right]\left\{ \begin{gathered} {f_{y1}} \hfill \\ {f_{y2}} \hfill \\ \end{gathered} \right\}$$

5 $$\left\{ \begin{gathered} {M_{x1}} \hfill \\ {M_{x2}} \hfill \\ \end{gathered} \right\}=\left[ \begin{gathered} \frac{l}{3}\;\;\;\frac{l}{6} \hfill \\ \frac{l}{6}\;\;\;\frac{l}{3} \hfill \\ \end{gathered} \right]\left\{ \begin{gathered} {m_{x1}} \hfill \\ {m_{x2}} \hfill \\ \end{gathered} \right\}$$

By performing inverse transformations on formulas (4) and formulas (5), then bring them into formula (3), it can be concluded that:

6 $$\left\{ \begin{gathered} {\sigma _{s1}} \hfill \\ {\sigma _{s2}} \hfill \\ \end{gathered} \right\}=\frac{1}{t}\left[ \begin{gathered} \frac{4}{l}\;\;\;\;\;\frac{{ - 2}}{l} \hfill \\ \frac{{ - 2}}{l}\;\;\;\;\frac{4}{l} \hfill \\ \end{gathered} \right]\left( {\left\{ \begin{gathered} {F_{y1}} \hfill \\ {F_{y2}} \hfill \\ \end{gathered} \right\}+\left\{ \begin{gathered} {M_{x1}} \hfill \\ {M_{x2}} \hfill \\ \end{gathered} \right\}} \right)$$

Generally, when a section of weld is divided into *n* elements, the node number is from 1 to *n*, the distance of each node on the welding line is ${l_1}$ to ${l_{n - 1}}$, and the structural stress equation can be written as:

7 $${\sigma _s}=\frac{1}{t}{L^{ - 1}}{F_{yn}}+\frac{6}{t}{M_{xn}}$$

where

8 $${L^{ - 1}}=\left[ {\begin{array}{*{20}{c}} {\left( {\frac{3}{{{l_1}}}+\frac{1}{{{l_1}+{l_2}}}} \right)}&{\frac{{ - 2}}{{{l_1}+{l_2}}}}&{\frac{1}{{{l_1}+{l_2}}}}&0& \ldots &0 \\ {\frac{{ - 2}}{{{l_1}+{l_2}}}}&{\frac{4}{{{l_1}+{l_2}}}}&{\frac{{ - 2}}{{{l_1}+{l_2}}}}&0& \ldots &0 \\ 0&{\frac{{ - 2}}{{{l_2}+{l_3}}}}&{\frac{4}{{{l_2}+{l_3}}}}&{\frac{{ - 2}}{{{l_2}+{l_3}}}}& \ldots &0 \\ 0&0& \ddots & \ddots & \ddots &0 \\ \vdots & \ddots & \ddots &{\frac{{ - 2}}{{{l_{n - 2}}+{l_{n - 1}}}}}&{\frac{4}{{{l_{n - 2}}+{l_{n - 1}}}}}&{\frac{{ - 2}}{{{l_{n - 2}}+{l_{n - 1}}}}} \\ 0& \ldots & \ldots &{\frac{1}{{{l_{n - 2}}+{l_{n - 1}}}}}&{\frac{{ - 2}}{{{l_{n - 2}}+{l_{n - 1}}}}}&{\left( {\frac{3}{{{l_{n - 1}}}}+\frac{1}{{{l_{n - 2}}+{l_{n - 1}}}}} \right)} \end{array}} \right]$$

### Master S-N curve method

**Fig. 2:**
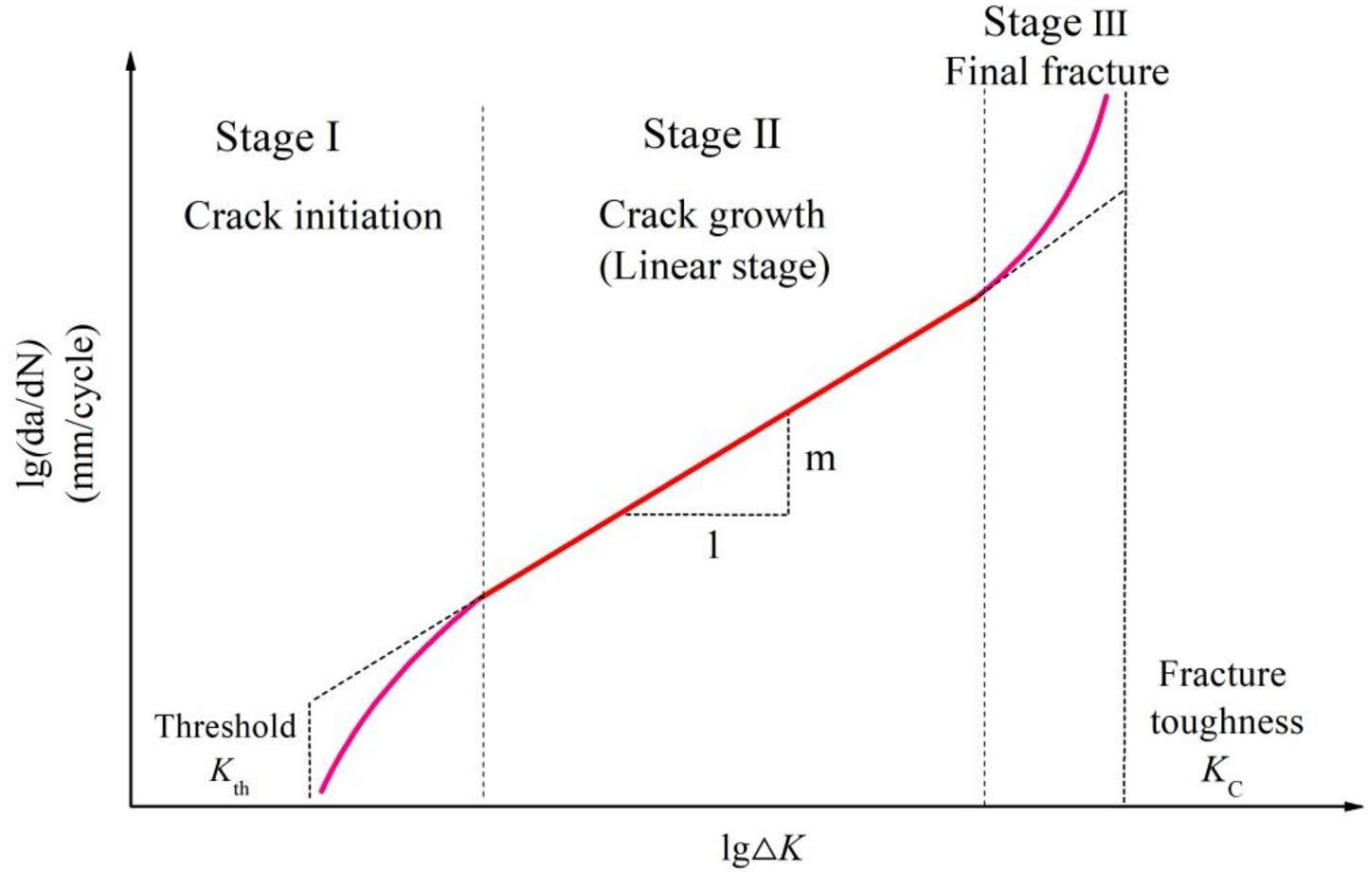
Schematic diagram of three-stage fatigue crack growth.

Fatigue crack growth can be divided into three stages: crack initiation stage, crack growth stage and final fracture stage^41^, as shown in Fig. 2. Paris crack growth rate can been unified into the following formula:

9 $$\frac{{\operatorname{d} a}}{{\operatorname{d} N}}=C{\left( {{M_{kn}}} \right)^n}{\left( {\Delta {K_n}} \right)^m}$$

Where *a* is crack length; *N* is number of endurance cycles; ${M_{kn}}$ is the stress strength factor amplification factor caused by the weld toe notch; *n* is the crack extension index of short crack extension stage, the empirical value is 2; *m* is the crack extension index of conventional Paris equation, the value is 3.6; *C* is crack extension parameter of the material; $\Delta {K_n}$ is the stress strength factor of the plate edge crack tip, and its value can be obtained by the formula (10).

10 $$\Delta K=\sqrt t \left[ {\Delta {\sigma _m}{f_m}\left( {a/t} \right)+\Delta {\sigma _b}{f_b}\left( {a/t} \right)} \right]$$

Where, ${f_m}\left( {a/t} \right)$ and ${f_b}\left( {a/t} \right)$ are the dimensionless weight functions determining the range of stress strength factors when membrane stress and bending stress alone, respectively. ${f_m}\left( {a/t} \right)$ and ${f_b}\left( {a/t} \right)$ are obtained by formula (11) and formula (12).

11 $${f_m}\left( {\frac{a}{t}} \right)=\frac{{\sqrt {2\tan \frac{{\pi a}}{{2t}}} \left[ {0.75+2\left( {\frac{a}{t}} \right)+0.37{{\left( {1 - \sin \frac{{\pi a}}{{2t}}} \right)}^3}} \right]}}{{\cos \frac{{\pi a}}{{2t}}}}$$

12 $${f_b}\left( {\frac{a}{t}} \right)=\frac{{\sqrt {2\tan \frac{{\pi a}}{{2t}}} \left[ {0.92+0.2{{\left( {1 - \sin \frac{{\pi a}}{{2t}}} \right)}^4}} \right]}}{{\cos \frac{{\pi a}}{{2t}}}}$$

By integrating formula (9), the prediction expression for fatigue life from short crack to penetrating plate is

13 $$N=\mathop \smallint \limits_{{\frac{a}{t} \to 0}}^{{a/t=1}} \frac{{t\operatorname{d} \left( {\frac{a}{t}} \right)}}{{C{{\left( {{M_{kn}}} \right)}^n}{{\left( {\Delta {K_n}} \right)}^m}}}=\frac{1}{C}{t^{1 - \frac{m}{2}}}{\left( {\Delta {\sigma _s}} \right)^{ - m}}I\left( r \right)$$

*I(r)* is a dimensionless function of the load bending ratio *r*, and its mathematical expression is

14 $$\left\{ \begin{gathered} I\left( r \right)=\mathop \smallint \limits_{{\frac{a}{t} \to 0}}^{{a/t=1}} \frac{{\operatorname{d} \left( {\frac{a}{t}} \right)}}{{{{\left( {{M_{kn}}} \right)}^n}{{\left[ {{f_m}(\frac{a}{t}) - r({f_m}(\frac{a}{t}) - {f_b}(\frac{a}{t}))} \right]}^m}}} \hfill \\ r=\frac{{\left| {\Delta {\sigma _b}} \right|}}{{\left| {\Delta {\sigma _s}} \right|}} \hfill \\ \end{gathered} \right.$$

In the welded structure, for the semi-elliptic crack propagation and a displacement controlled condition, the following formula can be obtained.

15 $$I{\left( r \right)^{\frac{1}{{\text{m}}}}}=2.1549{r^6} - 5.0422{r^5}+4.8002{r^4} - 2.0694{r^3}+0.561{r^2}+0.0097r+1.5426$$

From the above analysis, the equivalent structural stress expression is

16 $$\Delta S=\frac{{\Delta {\sigma _s}}}{{{t^{\left( {2 - m} \right)/2m}}I{{\left( r \right)}^{1/m}}}}$$

Combining formula (16), the constant in formula (13) needs to be corrected using a large amount of experimental data. The corrected formula can be expressed as

17 $$N= \Delta S/{C_d}{r ^{ - 1/h}}$$

Where ${C_d}$ and *h*are experimental constants, which can be obtained in reference^42^.

### The fatigue assessment method of BSI standard

**Fig. 3:**
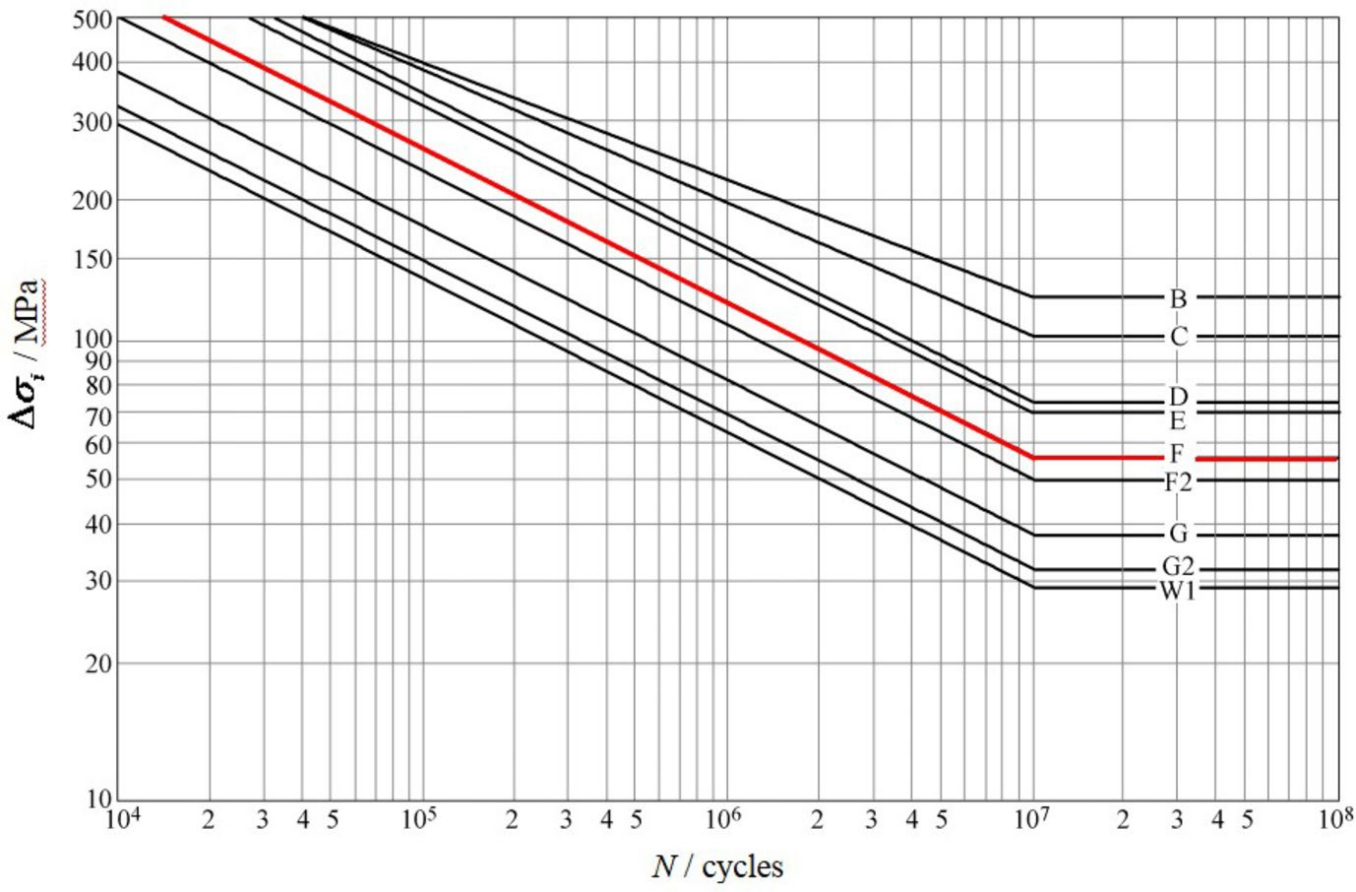
S-N curves of BSI standard.

“Guide to fatigue design and assessment of steel products”^9^ is the most representative of the anti-fatigue design and evaluation methods based on nominal stress, which is widely used for fatigue strength assessment of welded joints in railway vehicles, automobiles, aerospace, bridges, and other industries. The S-N curve in this standard was obtained through a large number of fatigue tests, and the S-N curve considers the effects of local stress concentration, maximum allowable discontinuity in size and shape, and welding process effects, etc^43^. This standard provides S-N curves at different levels, as shown in Fig. 3. The specific welding joint details and fatigue performance parameters can refer to this standard, the F-level parameters (*m* = 3, *S*_r_=40 MPa) of T-joint is used for the fatigue damage assessment of the high-ply cutter body in this paper.

where $\Delta {\sigma _i}$ is the stress range; *N* is the number of endurance cycles.

The fatigue resistance design and evaluation method of steel structures given in this standard is based on the Palmgren-Miner cumulative damage theory, and the damage ratio is defined as:

18 $$~\frac{{{n_i}}}{{{N_i}}}=\left\{ \begin{gathered} \frac{{{n_i}}}{{{{10}^7}}}{(\frac{{\Delta {\sigma _i}}}{{\Delta {\sigma _0}}})^m}\;\;\;\;\;(\Delta {\sigma _i}>\Delta {\sigma _0}) \hfill \\ \frac{{{n_i}}}{{{{10}^7}}}{(\frac{{\Delta {\sigma _i}}}{{\Delta {\sigma _0}}})^{m+2}}\;\;\;(\Delta {\sigma _i} \leqslant \Delta {\sigma _0}) \hfill \\ \end{gathered} \right.$$

where ${n_i}$ is the number of cycles with stress range $\Delta {\sigma _i}$ in the load spectrum; ${N_i}$ is the total number of cycles that lead to structural damage when the stress range is $\Delta {\sigma _i}$; $\Delta {\sigma _0}$ is the stress range when the evaluation point is $1 \times {10^7}$; *m* is the slope of the S-N curve.

## Applicability of master S-N curve approach in high-ply cutter

### The structure of high-ply cutter body and loading method

**Fig. 4:**
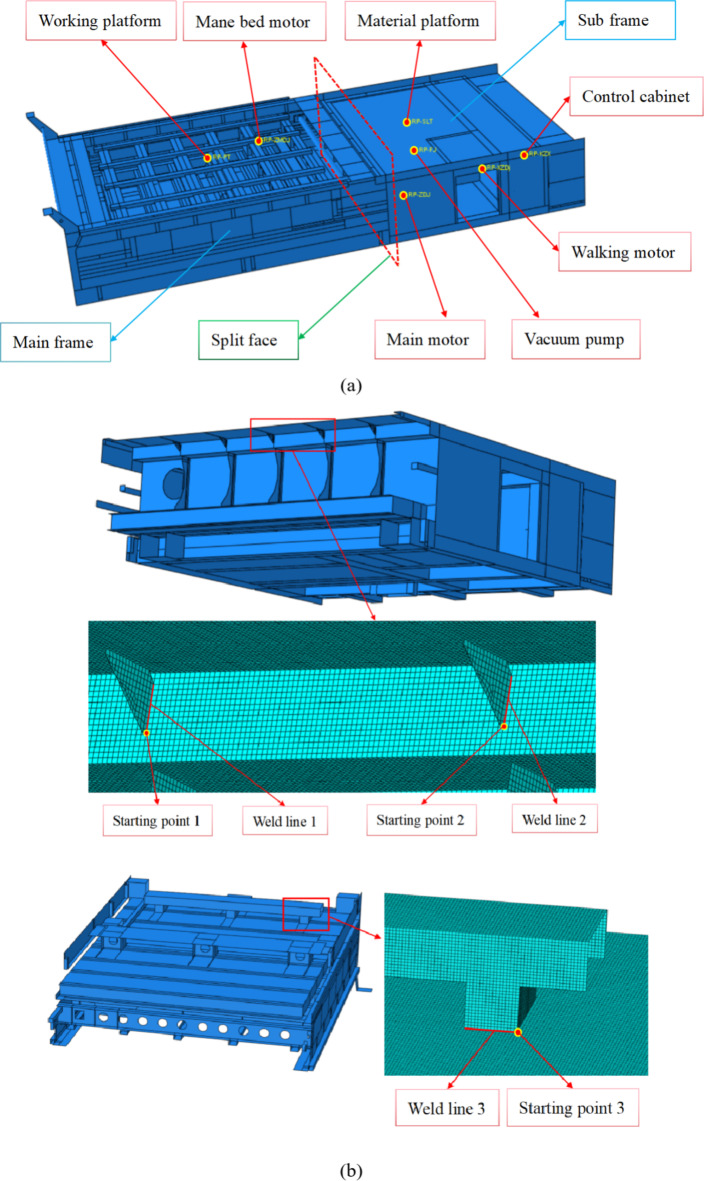
High-ply cutter body. (**a**) Main structures and installation positions of key components and (**b**) Weld line and welding starting point of key welds.

The high-ply cutter body is all-steel welded structure, it mainly consists of the main frame and sub frame, which is shown in Fig. 4(a). Meanwhile, the split face, the main platform, the control cabinet, and the installation positions of vacuum pump, main motor, mane bed motor, walking motor are shown in Fig. 4(a).

Based on design experience and finite element simulation results of similar cutter bodies, the fatigue strength analysis of the three key welds is focused in this article, their weld lines and welding starting points are shown in Fig. 4(b). In order to better simulate the stress state at the weld, the contour of the weld at this location needs to be considered in FEM model. Taking into account the computational complexity, accuracy, and actual situation of the entire structure, the modeling quality control of the three welds meets the requirements of ASME standard.

The material used for the cutter body is Q235B, and its chemical compositions and mechanical properties are shown in Tables 1 and 2, respectively^44^.

**Table 1:** Chemical compositions of Q235B.

Element (%)	C	Si	Mn	*P*	S
Q235B	≤ 0.20	≤ 0.35	≤ 1.40	≤ 0.045	≤ 0.045

**Table 2:** Mechanical properties of Q235B.

Performance index	Lower yield strength(MPa)	Tensile strength (MPa)	Elongation after fracture (%)	Impact property (J)
Q235B	235	370 ~ 500	26	27

The load position and constraint position of the finite element model are determined based on the actual working cavity vacuum pressure and the weight of each main functional component. The load under normal cutting conditions is shown in Table 3, and the corresponding force positions are shown in Fig. 4(a).

**Table 3:** The load under normal cutting conditions.

Number	Load name	weight
1	Vacuum pressure	0.025 (MPa)
2	Working platform	500 (kg)
3	Mane bed motor	60 (kg)
4	Material platform	150 (kg)
5	Control cabinet	200 (kg)
6	Main motor	300 (kg)
7	Vacuum pump	200 (kg)
8	Walking motor	26 (kg)

### Stress comparison analysis

**Fig. 5:**
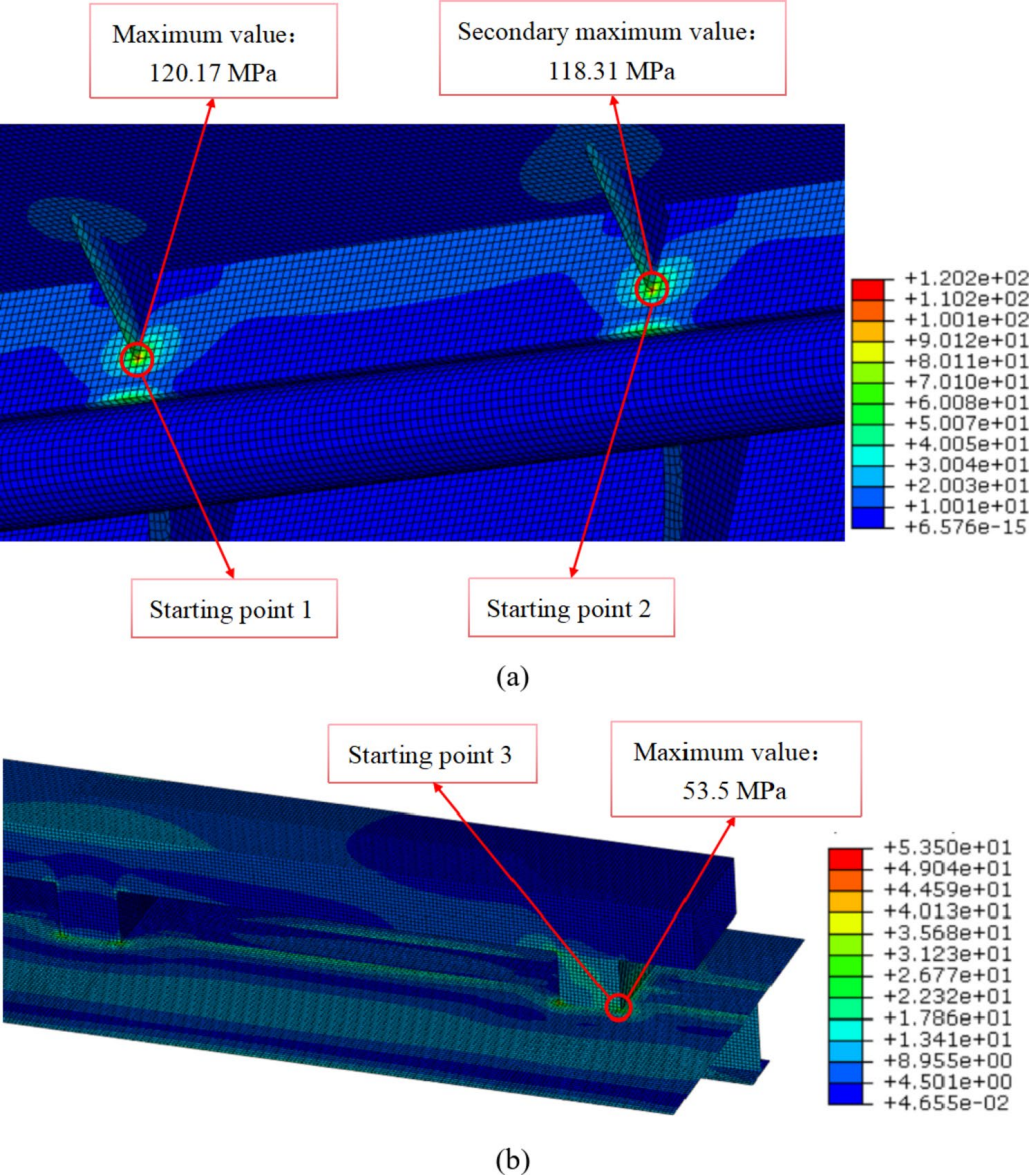
The local maximum stresses of three starting points. (**a**) point 1 and point 2 and (**b**) point 3.

**Fig. 6:**
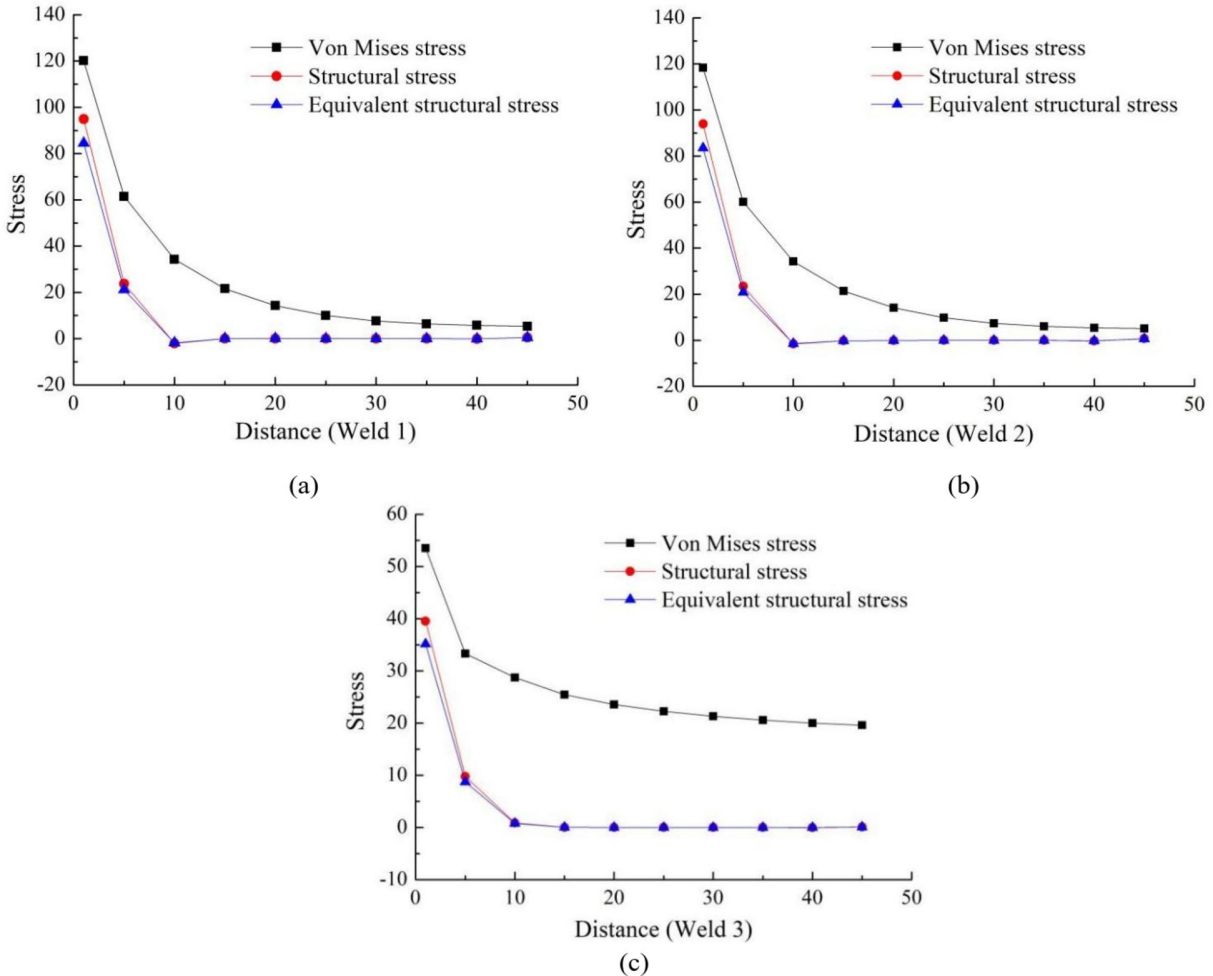
The stresses distribution of welds. (**a**) Weld 1, (**b**) Weld 2 and (**c**) Weld 3.

According to the finite element simulation results of cutter body, the Von Mises stresses of three starting points are shown in Fig. 5. Meanwhile, the Von Mises stresses of the three welds are extracted, and the structural stresses and equivalent structural stresses of the three welds are calculated, which are shown in Fig. 6. According to the Fig. 6, The following conclusions can be drawn:

1. On the whole, the stresses variation trends of the three welds are basically the same, gradually decreasing from the starting point. According to the positions of the three welds in Fig. 4(b), it can be seen that the starting points of three welds are all stiffness mutation positions. Therefore, the stiffness mutation is the main reason for the high stresses of the welds, especially for weld 1 and weld 2. In addition, the stress distribution of the three welds is that Von Mises stress is the highest, followed by structural stress, and equivalent structural stress is the lowest.
2. According to Fig. 6 (a) and Fig. 6 (b), the three types of stresses in weld 1 and weld 2 are relatively similar, due to the fact that the external dimensions and functions of the two support plates are the same and the distance is relatively close. Meanwhile, the stress of the two welds is relatively high at the starting point, which requires special attention in fatigue strength assessment.
3. According to Fig. 6 (c), compared to the stress of weld 1 and weld 2, the stress of weld 3 is relatively low. However, during the loading and unloading of the cutter body, the rectangular steel is an important connector connected to the crane cable, and its working condition is relatively harsh. Therefore, the point of maximum stress on weld 3 needs to be paid special attention.

### Fatigue damage analysis

**Fig. 7:**
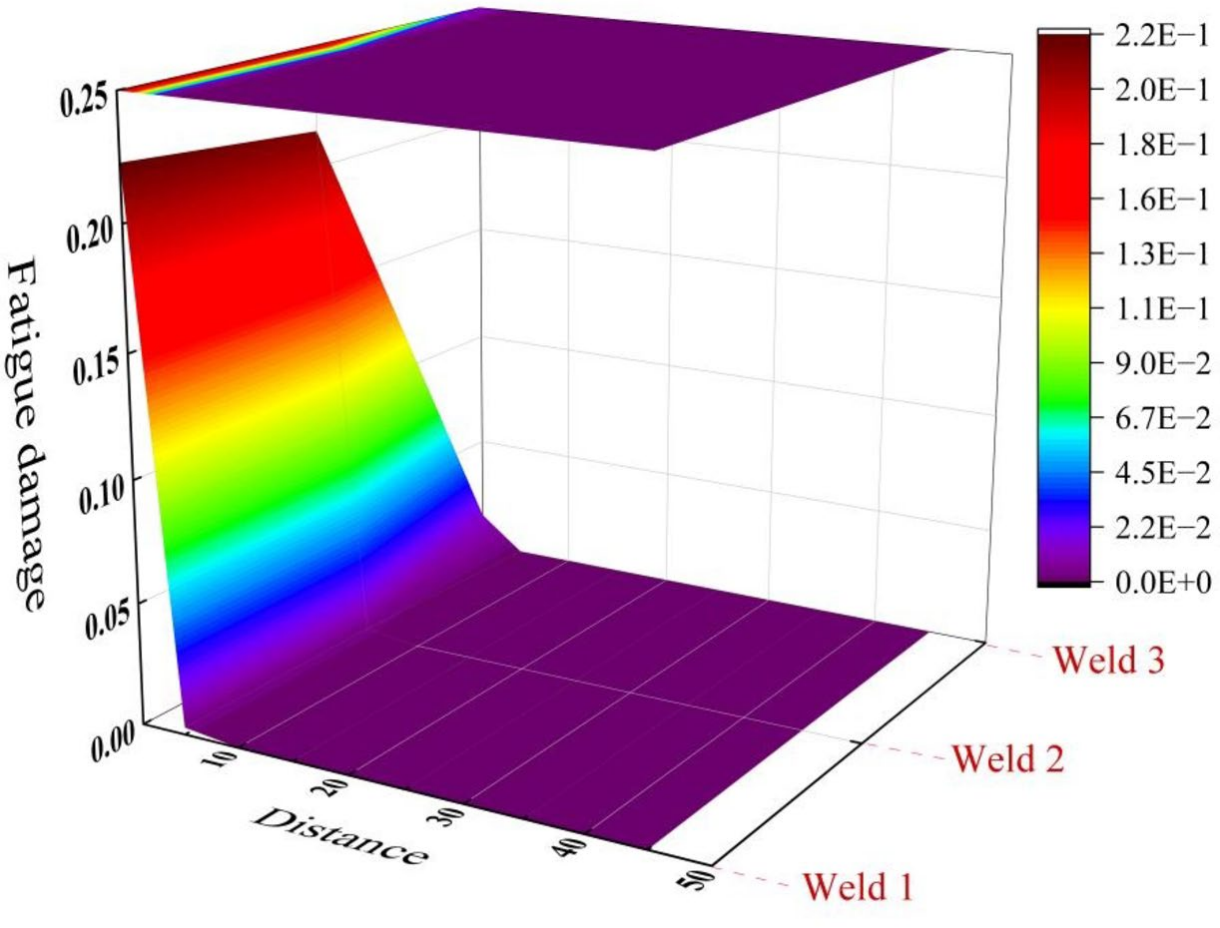
The fatigue damage of three welds.

According the master S-N curve method, the equivalent structural stress of the three welds in Fig. 6 is substituted into formula (16), and the fatigue damage of the three welds can be obtained, as shown in Fig. 7.

According to the Fig. 7, the fatigue damage of the three welds did not exceed 0.25, especially when the distance exceeded 5 mm, the fatigue damage was below 10^−4^, which meets the design requirements. In addition, the fatigue damages of weld 1 and weld 2 at starting points is 0.23 and 0.22, respectively, and weld 1 is slightly larger than weld 2. It indicates that the support plate near the air outlet is required to withstand a greater negative pressure.

## Discussion

### Dynamic stress test

In order to verify the applicability of the main S-N curve method and traditional fatigue damage assessment methods in the anti-fatigue design of cutters, the dynamic stress test was conducted.

**Fig. 8:**
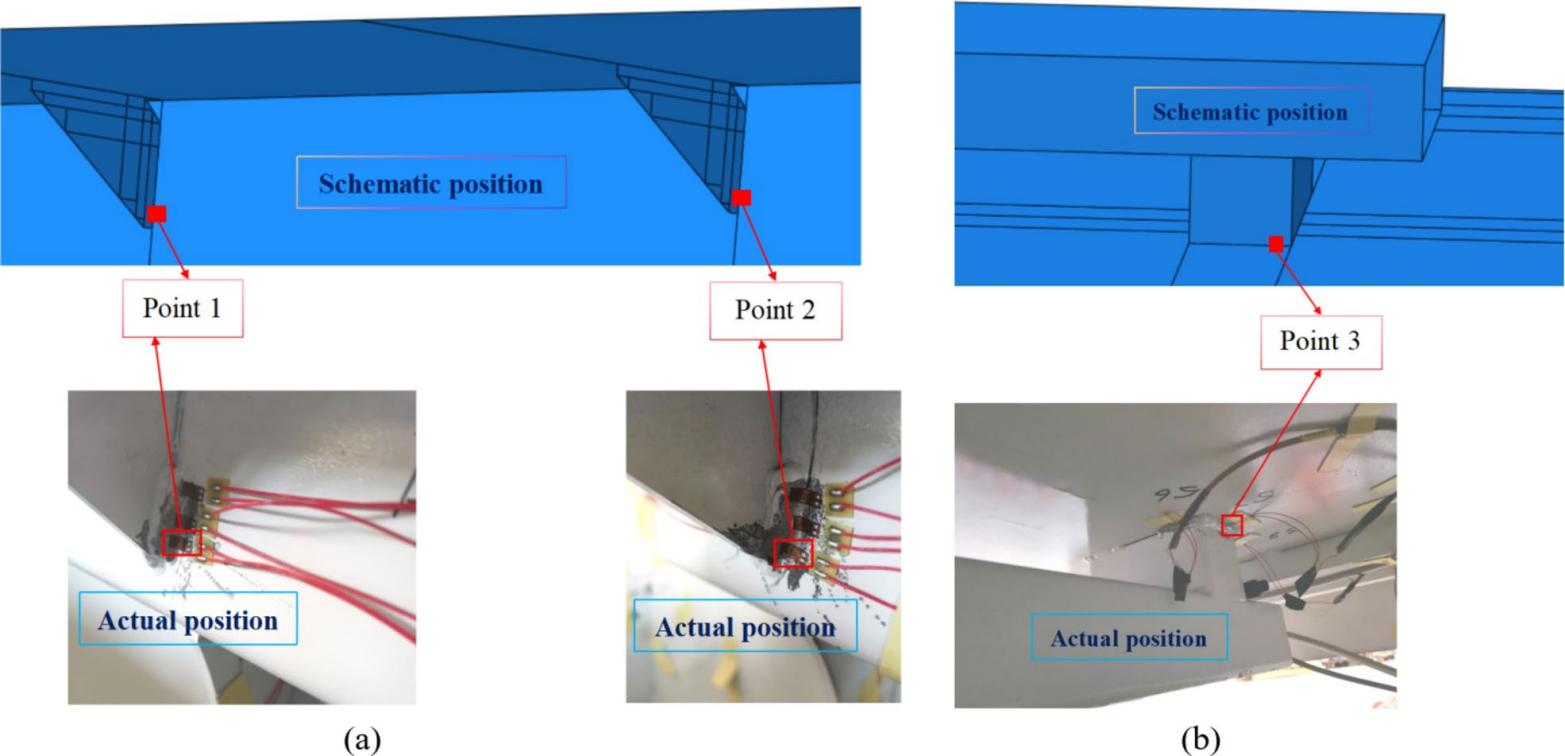
The schematic diagrams and measuring points’ positions (**a**) Point 1, Point 2 and (**b**) Point 3.

According to the calculation results of finite element simulation, in order to avoid the welding arc starting position, all three measuring points are at a distance of 5 mm from the starting point, as shown in Fig. 8. For ease of description, “measuring point” is abbreviated as “point”. The 120 strain gauges are arranged in a full bridge to eliminate the influence of environmental temperature and test wire resistance on the data signal. Meanwhile, in order to ensure the integrity of the experimental data, the sampling frequency was set to 10^5^ Hz. The experimental duration is 10 days.

### Stress amplitude spectrum

**Fig. 9:**
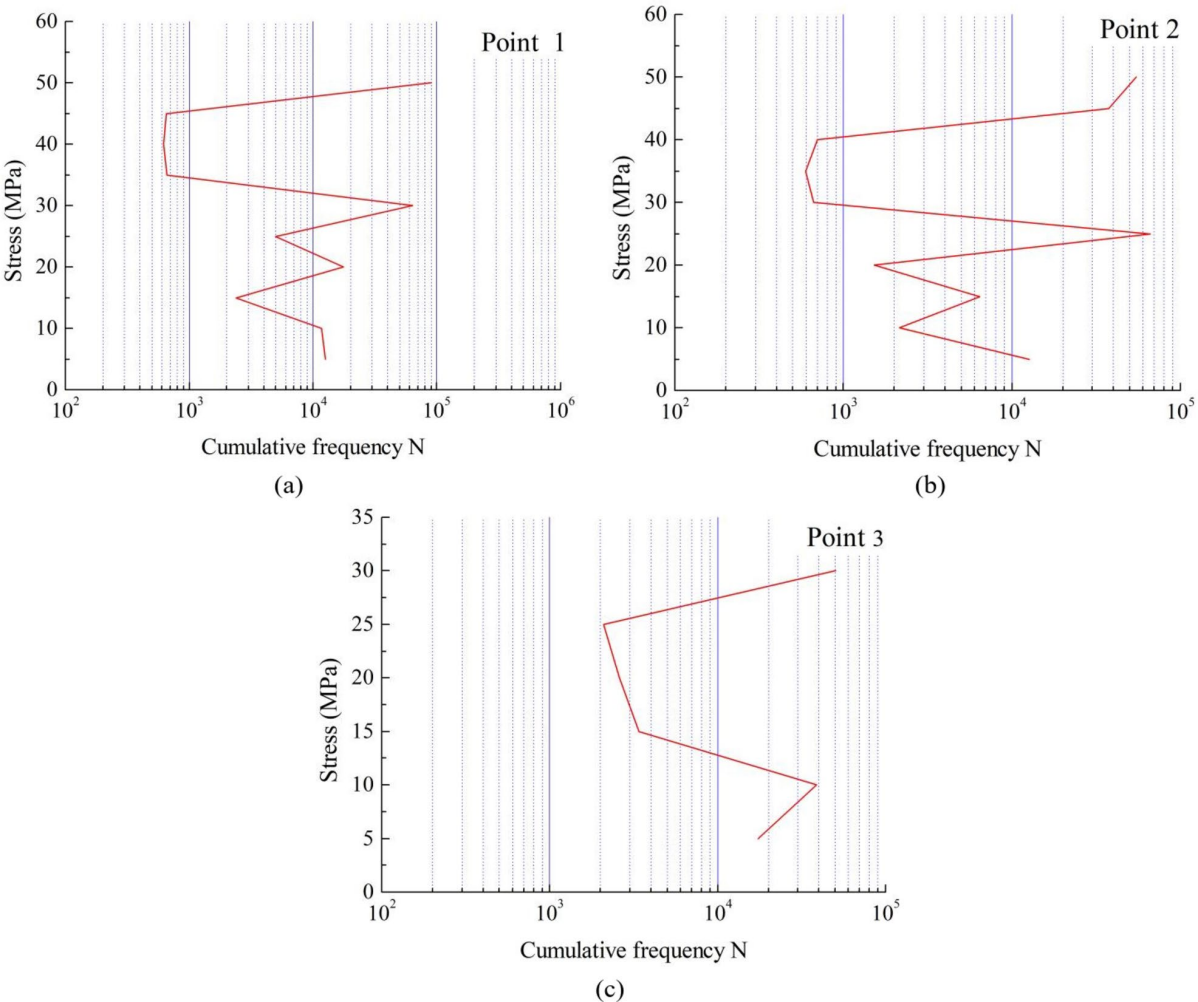
The stress spectrum curves of (**a**) Point 1, (**b**) Point 2 and (**c**) Point 3.

The measured data of the cutter body have been treated by glitch signal processing, zero drift processing removal, and interference filtering. Then, the rainflow counting method is used to perform mathematical statistics on the stress time history, and an equivalent 1-year stress amplitude spectrum is compiled. The influence of small amplitude stress is ignored during data processing, and the stress amplitude spectrum of three key measuring points are finally obtained, as shown in Fig. 9.

According to the Fig. 9, The following conclusions can be drawn:

1. On the whole, the three points all showed the maximum cumulative frequency at two specific stress points. The reason for this is that according to the specific cutting fabric and thickness, there are two working states in the high-ply cutter during operation, namely two different vacuum pressure. It results in the main cumulative frequency being concentrated on two specific stresses.
2. According to Fig. 9(a) and Fig. 9(b), we can see that the variation trends of the two stress amplitude spectrum are similar, but the overall stress amplitude spectrum of point 2 is smaller than that of point 1. Figure 9(b) is equivalent to moving Fig. 9(a) downward and to the right as a whole. This phenomenon indicates that not only is the overall stress at point 2 smaller than that at point 1, but the number of cycles at the same stress is also smaller than that at point 1.
3. According to Fig. 9(c), compared to points 1 and 2, the overall stress at point 3 is smaller and its cumulative frequency is similar to point 2, but the minimum value is slightly higher than the other two points. It can be seen that the dynamic stress of point 3 is mainly concentrated between 10 MPa-30 MPa in actual work, and indicating that the fatigue damage at this position is relatively small under normal working conditions.

### Fatigue damage evaluation

According to the finite element simulation results of cutter body, the Von Mises stresses of the three key points are extracted, and combining the load spectrum of high-ply cutter, S-N curve and Miner’s linear cumulative damage theory, the fatigue damages of three key points with Von Mises stresses have been calculated, as shown in Table 4. According to the stress amplitude spectrum shown in Fig. 9, S-N curve in the BS7608 standard and Miner’s linear cumulative damage theory, the design life of high-ply cutter is 30 years, so the fatigue damage corresponding to the measured stress spectrum is equivalent to 30 years of fatigue damage, as shown in Table 4. In addition, fatigue damages of three key points with master S-N curve were extracted from Fig. 7, as shown in Table 4.

**Table 4:** Fatigue damage at three points.

Measuring point	1	2	3
Master S-N curve	0.223	0.215	1.44E-02
Von Mises stress method	0.217	0.155	1.17E-03
Measured stress spectrum	0.193	0.168	1.23E-02

In practical engineering applications, safety margins must be considered in the anti-fatigue design of welded structural components. According to reference^45^, the fatigue damage of welded structural components generally should not exceed 0.5. According to Table 4, The fatigue damage of the three measuring points calculated by the three methods is all below 0.5, and it indicates that the design of the cutter body is relatively reasonable.

According to Table 4, the fatigue damage of the three points calculated by master S-N curve is higher than that obtained by the other two methods, which indicates that master S-N curve is more safety in evaluating the fatigue damage of the high-ply cutter body. Due to the fact that all three measuring points are 1 mm away from the arc starting points of the welds, and the points of both simulation methods are located at the starting point (which are stiffness mutation points) of the weld, which leads to higher fatigue damage obtained by simulation methods than measured. In addition, in the fatigue damage of Von Mises stress method, the stresses at point 1 and point 2 are similar, but there is a significant difference in fatigue damage values. The reason is that the load cycles at point 2 is smaller than that at point 1.

## Conclusions

1. The structural stress method and master S-N curve method is first applied to fatigue damage assessment of the high-ply cutter body, and the calculation process is systematically given in this paper.
2. The fatigue damages obtained by the master S-N curve, Von Mises stress method, measured stress spectrum did not exceed 0.5, and the fatigue damage at measuring point 1 and point 2 was around 0.2. In the subsequent structural optimization, the positions need to be paid special attention.
3. By comparing and analyzing the fatigue damages obtained by three methods (the master S-N curve, Von Mises stress method, measured stress spectrum), it can be seen that the fatigue damage calculated by the master S-N curve method is the safest. This indicates that the master S-N curve method is feasible for evaluating the fatigue damage of high-ply cutter body, and it can provide a basis for the anti-fatigue design and structural optimization of high-ply cutters.

## Data Availability

The datasets used and/or analysed during the current study available from the corresponding author on reasonable request.
